# Approximate Mortality Risks between Hyperuricemia and Diabetes in the United States

**DOI:** 10.3390/jcm8122127

**Published:** 2019-12-03

**Authors:** Po-Hsun Chen, Yu-Wei Chen, Wei-Ju Liu, Ssu-Wei Hsu, Ching-Hsien Chen, Chia-Lin Lee

**Affiliations:** 1 Department of Internal Medicine, Taichung Veterans General Hospital, Chiayi and Wanqiao Branch, Chiayi 60090, Taiwan; mbom101@gmail.com; 2 Department of Internal Medicine, Taipei Veterans General Hospital, Yuanshan and Suao Branch, Yilan 26444, Taiwan; cyw730505@gmail.com; 3 Department of Medical Research, Taichung Veterans General Hospital, Taichung 40705, Taiwan; u103092002@cmu.edu.tw; 4 Division of Nephrology, Department of Internal Medicine, University of California Davis, Davis, CA 95616, USA; suwhsu@ucdavis.edu; 5 Division of Endocrinology and Metabolism, Department of Internal Medicine, Taichung Veterans General Hospital, Taichung 40705, Taiwan; 6 Department of Medicine, School of Medicine, National Yang-Ming University, Taipei 11221, Taiwan

**Keywords:** hyperuricemia, diabetes, cardiovascular disease, mortality risk, uric acid

## Abstract

**Aim**: This study aimed to compare mortality risks across uric acid (UA) levels between non-diabetes adults and participants with diabetes and to investigate the association between hyperuricemia and mortality risks in low-risk adults. **Methods**: We analyzed data from adults aged >18 years without coronary heart disease and chronic kidney disease (*n* = 29,226) from the National Health and Nutrition Examination Survey (1999–2010) and the associated mortality data (up to December 2011). We used the Cox proportional hazards models to examine the risk of all-cause and cause-specific (cardiovascular disease (CVD) and cancer) mortality at different UA levels between adults with and without diabetes. **Results**: Over a median follow-up of 6.6 years, 2069 participants died (495 from CVD and 520 from cancers). In non-diabetes adults at UA ≥ 5 mg/dL, all-cause and CVD mortality risks increased across higher UA levels (*p*-for-trend = 0.037 and 0.058, respectively). The lowest all-cause mortality risk in participants with diabetes was at the UA level of 5–7 mg/dL. We set the non-diabetes participants with UA levels of <7 mg/dL as a reference group. Without considering the effect of glycemic control, the all-cause mortality risk in non-diabetes participants with UA levels of ≥7 mg/dL was equivalent to risk among diabetes adults with UA levels of <7 mg/dL (hazard ratio = 1.44 vs. 1.57, *p* = 0.49). A similar result was shown in CVD mortality risk (hazard ratio = 1.80 vs. 2.06, *p* = 0.56). **Conclusion**: Hyperuricemia may be an indicator to manage multifaceted cardiovascular risk factors in low-risk adults without diabetes, but further studies and replication are warranted.

## 1. Introduction

Studies in adults of various ethnicities reported that gout was associated with a higher risk of death from all causes and cardiovascular disease (CVD) [1,2]. Hyperuricemia is an important factor of gout, but it is to be determined whether hyperuricemia is a causal risk factor or just a marker for mortality. In the United States, hyperuricemia prevalence was >20% in both men and women (definition of hyperuricemia: Serum urate level of >7.0 mg/dL in men and >5.7 mg/dL in women) [3], even higher than diabetes prevalence. Asymptomatic hyperuricemia was reported to be associated with systemic inflammatory markers [4] and could even predict higher C-reactive protein levels when tested during a period of three years [5]. It has been demonstrated that gout may induce refractory inflammation that contributes to CVD [6], similar to inflammation that induces atherosclerosis change in rheumatoid arthritis [7] and systemic lupus erythematosus [8]. However, it was not evident that urate-lowering therapy (ULT) could sufficiently lower CVD risk [9] or reduce the risk of decline of renal function in patients with chronic kidney disease (CKD) and hyperuricemia [10,11].

Mortality trends have changed in diabetes patients and there was no difference in cardiac death between participants with and without diabetes during 2005–2010 in the United States [12]. However, the proportion of cardiac mortality in people without diabetes did not decrease as it did with the diabetes population [12]. Hyperuricemia, which can be affected by diet, life habits, and genetic factors, is a problem similar to hyperglycemia. Although hyperuricemia is a potential risk factor/marker for mortality, it is not suggested to be controlled aggressively like diabetes, hyperlipidemia, or hypertension in clinical guidelines [13,14].

In this study, we compared mortality risks between participants with and without diabetes across different uric acid (UA) levels. By comparing mortality risks between non-diabetes adults and participants with diabetes, we aimed to investigate the potential role of hyperuricemia associated with mortality risks in adults without diabetes. 

## 2. Materials and Methods

### 2.1. Data Source and Study Population

The National Health and Nutrition Examination Survey (NHANES) data was constructed by the National Center for Health Statistics (NCHS). The NHANES examinations included a standardized medical examination and questionnaires to address different health-related records. We analyzed data from a variety of records within NHANES (1999–2010, *n* = 62,160) and their linked National Death Index mortality data (through 31 December 2011). The participants without data of serum UA levels were excluded. Furthermore, the participants with CKD or coronary heart disease (CHD) were excluded. The definition of CKD was impaired glomerular filtration rate (GFR) of <60 ml/min/1.73 m^2^. Estimated GFR was calculated using the chronic kidney disease epidemiology collaboration (CKD-EPI) equation [15]. The participants were classified into having CHD if they answered yes to the following question: Have you ever been told you had coronary heart disease? In total, 29,226 participants aged ≥18 years were included (Supplementary Figure S1).

### 2.2. Definition of Diabetes

There are only 29,174 participants with glycated hemoglobin (HbA1c) data enrolled in this study. The participants who self-reported a physician’s diagnosis of diabetes or self-reported taking insulin or diabetic pills were categorized as having diabetes mellitus (DM). Overall, 2506 participants were classified as having diabetes. The type of DM was not accounted for adjustment or exclusion. 

### 2.3. Mortality Outcomes

Mortality outcomes of interest in this study include all-cause mortality, cardiovascular death, and cancer death, based on ICD-10 (10th revision of the International Statistical Classification of Diseases and Related Health Problems) codes defined in NHANES. To identify causes of death in participants, the specific codes were the following: I00–I09, I11, I13, I20–I51, and I60–I69 were categorized as cardiovascular death, while the codes of C00–C97 were causes of death from malignant neoplasms (cancer death). Mortality status for NCHS survey participants was ascertained primarily through probabilistic record matching with the National Death Index (NDI) death certificate records [16].

### 2.4. Statistical Analysis

We utilized chi-square and analysis of variance (ANOVA) tests to examine significant differences in baseline demographics and characteristics across levels of uric acid and diabetes status. Cox proportional hazards regression models were used to compare the hazard ratios (HRs) and 95% CI (confidence interval) for the association of UA levels with all-cause, cardiovascular, and cancer mortality, separately in participants with and without DM. Additionally, we set the non-diabetes participants with UA levels of 5–7 mg/dL as the reference, which allowed mortality risks at every UA category in non-diabetes and diabetes groups to be compared. Similarly, different UA categorizations were set (<7, 7–9, ≥9, and <7, ≥7) in order to compare different mortality risks across UA levels between non-diabetes and diabetes groups. In addition, age, sex, race/ethnicity, body mass index (BMI), high-density lipoprotein cholesterol (HDL-C), systolic blood pressure (SBP), creatinine, and smoking state were adjusted in all survival analyses. All variables were from baseline data. Due to the complex survey design of the NHANES study, all analyses were adequately weighted to represent the US population. The weighted data were calculated according to analytic guidelines [17]. The un-weighted HRs were also presented as sensitivity analysis. All analyses were conducted using the Statistical Analysis System survey procedures (SAS version 9.4, 2013, Cary, NC, USA). *p*-values < 0.05 were considered statistically significant.

## 3. Results

To determine if elevated levels of uric acid are a risk factor for diabetes mellitus, we analyzed uric acid levels in both diabetic and non-diabetic adults. Table 1 and Table 2 document the characteristics of the subjects according to diabetic status and uric acid level at baseline, respectively. In the overall population, non-diabetes participants were associated with younger age (43.4 ± 0.2 vs. 56.7 ± 0.4, *p* < 0.001), lower prevalence of cancers (7.1% vs. 12.5%, *p* < 0.001), and hypertension (23.2% vs. 61.2%, *p* < 0.001), lower BMI (27.9 ± 0.1 vs. 32.5 ± 0.2, *p* < 0.001), lower SBP (121 ± 0.2 mmHg versus 131 ± 0.7 mmHg, *p* < 0.001), higher HDL-C (53.2 ± 0.2 mg/dL vs. 48.2 ± 0.4 mg/dL, *p* < 0.001), higher total cholesterol (199.6 ± 0.4 mg/dL vs. 196.9 ± 1.5 mg/dL, *p* < 0.001), and triglyceride (143.5 ± 1.2 mg/dL vs. 205.2 ± 7.1 mg/dL, *p* < 0.001). In non-diabetes participants, there were older age, higher BMI, higher prevalence of hypertension, lower HDL-C, higher total cholesterol, higher triglyceride, higher fasting blood glucose, and higher serum creatinine levels across higher UA categories (Table 2). Diabetes participants, however, consisted of more non-Hispanic Black participants, and were associated with older age, higher BMI, lower HDL-C, higher triglyceride, higher fasting blood glucose, and higher serum creatine levels across higher UA categories. In both the non-diabetes and diabetes groups, the cancer prevalence was insignificantly different across UA categories (*p*-value = 0.31 and 0.91, respectively).

The median follow-up period was 6.6 years. Among those participants who died (*n* = 2069, 7.9 per 1000 person-years), 495 (23.9%) died due to CVD, and 520 (25.1%) died due to cancer. Table 3 shows the adjusted un-weighted and weighted all-cause and cause-specific mortality risks across UA levels, separately for adults without diabetes and with diabetes. Different models are shown in Supplementary Table S1. For non-diabetes persons, when serum UA levels are above 5 mg/dL, the weighted HRs for all-cause mortality and CVD mortality increase significantly across higher UA levels (*p*-for-trend = 0.003 and 0.058, respectively). Among participants with diabetes, the lowest weighted all-cause mortality risk is at the UA level of 5–7 mg/dL. Regarding CVD mortality risk in participants with diabetes at different UA levels, no significant difference is found between UA levels of <5 mg/dL and 5–7 mg/dL, but the highest HR (2.53, 95% CI 1.18–5.41) is at UA levels of 7–9 mg/dL, when compared to UA levels of 5–7 mg/dL. Compared to the mortality risk at UA level of 5–7 mg/dL in non-diabetes adults, HRs at every UA category in non-diabetes and diabetes groups are shown in Table 4 and Supplementary Table S2. At UA levels of 5–7 mg/dL, CVD mortality risk is significantly higher (HR = 2.25, 95% CI 1.25–4.06) in participants with diabetes, but all-cause and cancer mortality risks are not. The significantly higher HRs for all-cause mortality risk in non-diabetes adults at UA levels of 7–9 mg/dL and ≥9 mg/dL are 1.40 and 2.35, respectively. In participants with diabetes, HRs for all-cause mortality risk at UA levels of <5 mg/dL, 7–9 mg/dL, and ≥9 mg/dL are significantly higher at 2.01, 2.87, and 2.79, respectively. CVD mortality risk in non-diabetes adults with UA levels of ≥9 mg/dL approximates the risk at UA levels of 7–9 mg/dL in diabetes patients (HR = 5.07 versus 4.99) and is even higher than those with diabetes and UA levels of 5–7 mg/dL (HR = 5.07 versus 2.25). However, the HRs for cancer-specific mortality did not differ significantly across UA levels among non-diabetes and diabetes participants. 

**Figure 1:**
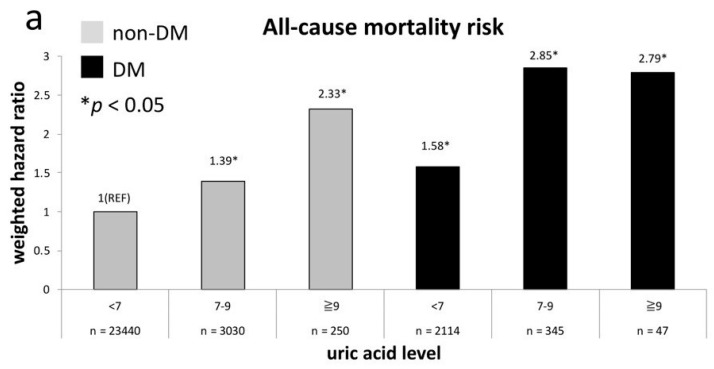

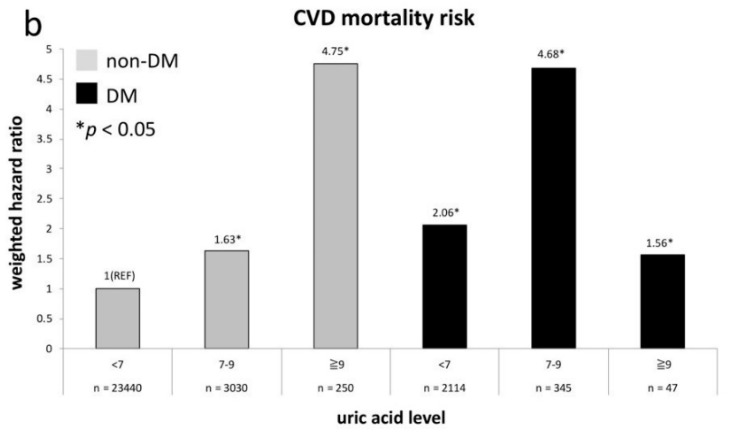
All-cause (**a**) and CVD (**b**) mortality risks at every uric acid (UA) category compared to non-diabetes mellitus (DM) participants with UA level of <7 mg/dL.

Figure 1a shows the weighted all-cause mortality risk after adjustment across UA levels in both non-diabetes and diabetes groups. It indicates that all-cause mortality risks in non-diabetes adults with UA levels of 7–9 mg/dL and ≥9 mg/dL are equivalent to the risk in those with diabetes and a UA level of <7 mg/dL (*p* = 0.33 and 0.17, respectively). The non-diabetes participants with a UA level of ≥9 mg/dL even have approximate risk to participants with diabetes with a UA level of 7–9 mg/dL or ≥9 mg/dL (*p* = 0.49 and 0.72, respectively). Figure 1b exhibits the comparison of weighted risk of CVD death after adjustment across UA levels between non-diabetes and diabetes groups. The CVD mortality risk in non-diabetes participants with a UA level of ≥9 mg/dL is comparable to the risk in those with diabetes and UA levels of 7–9 mg/dL (*p* = 0.98). The CVD mortality risks in non-diabetes participants with UA levels of 7–9mg/dl and diabetes participants with UA level of <7 mg/dl are nearly the same (HR = 1.63 versus 2.06, *p* = 0.31). Both groups have a significantly higher risk than non-diabetes participants with a UA level of <7 mg/dL (*p* = 0.008 and 0.002, respectively). 

**Figure 2:**
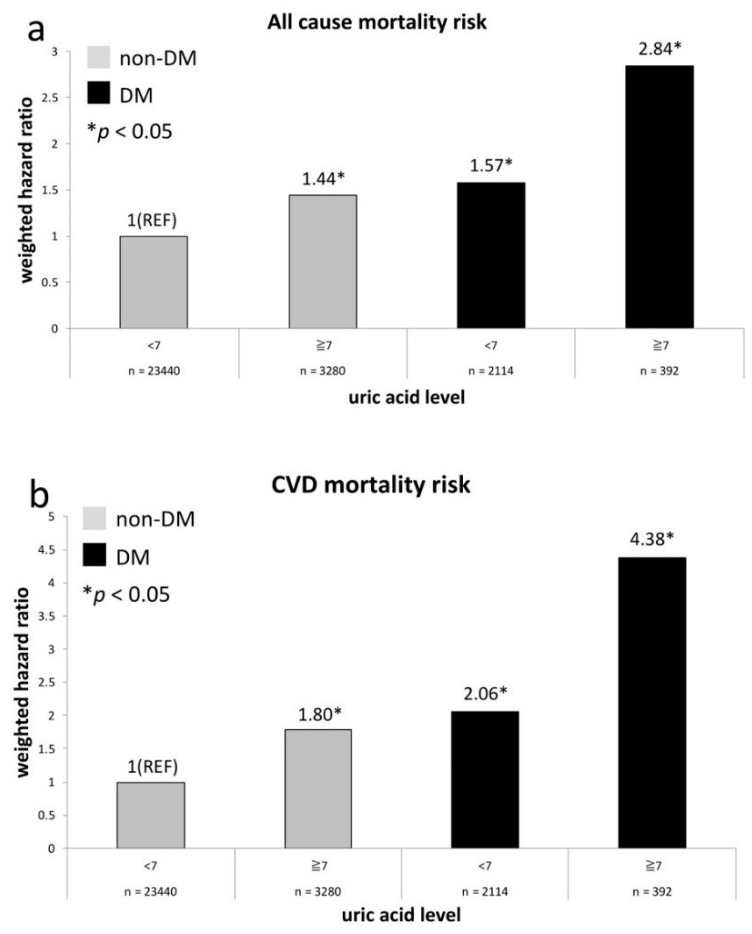
All-cause (**a**) and CVD (**b**) mortality risks between diabetic and non-diabetic groups compared with non-DM participants of UA level <7 mg/dL.

The HRs regarding risks of all-cause death and CVD death in relation to UA levels (UA < 7 mg/dL and ≥7 mg/dL) and history of DM at baseline are exhibited in Figure 2. The comparative reference is also non-diabetes participants with a UA level of <7 mg/dL. We demonstrate that all-cause mortality risks in another three groups are all significantly higher than the reference group (Figure 2a) and the risk is equivalent between non-diabetes adults with a UA level of ≥7 mg/dL and diabetes adults with a UA level of <7 mg/dL (*p* = 0.48). Figure 2b exhibits the similar results in terms of CVD mortality risks. 

## 4. Discussion

In this study, all participants were without chronic kidney disease or coronary heart disease at baseline. The results demonstrate that the mortality risks in non-diabetes adults are significantly higher at UA level of ≥7.0 mg/dL and all-cause and CVD mortality risks in non-diabetes participants with a UA level of ≥9.0 mg/dL are approximate to, or even higher than, those with diabetes and a UA level of <9.0 mg/dL. Hyperuricemia was associated with a statistically significant increased risk of CVD and all-cause mortality but did not exhibit a significant increase in terms of cancer mortality risk. Similar results have been reported [18,19], but a comparison between hyperuricemia and diabetes status is not yet elucidated. In the present study, Table 4 and Figure 1 and Figure 2 show the joint effects of DM and different UA level.

We found that the trend of all-cause mortality risk across higher UA levels is similar to CVD mortality risk in non-diabetes adults. However, the major difference between the current study and other reports from South Korea [20] and Japan [21] are that both the Asian data show a U-shaped association between mortality risks and UA level. The lowest studied UA levels were further lower than the present study. Furthermore, diabetic status was not addressed in the two studies. Low UA level may be associated with malnutrition [22] and weak antioxidant protection from endothelial injury and oxidative stress [23,24]. The more critical issue is whether non-diabetes adults with hyperuricemia have a mortality risk comparable to that of diabetes adults. If this is true, it suggests a similar baseline risk of CVD mortality, and management of cardiovascular risk factors in adults with hyperuricemia may be similar to diabetes patients. Our data indicate all-cause and CVD mortality risks elevated across UA levels in non-diabetes adults, but the U-shaped relationship between UA level and all-cause mortality is reflected in the participants with diabetes (Table 3). It is still debated whether hyperuricemia is an independent risk factor for CVD or merely associated with other risk factors, including hypertension [25], renal disease [26], hyperlipidemia [27], and diabetes [28]. In patients with gout, ULT would be cost-effective [22]. In adults with asymptomatic hyperuricemia, routine ULT is not recommended in current guidelines [29,30]. In the Losartan Intervention For Endpoint reduction in hypertension (LIFE) trial, major CV protection was derived from inhibition of the renin-angiotensin-aldosterone system, despite the UA-lowering effect of Losartan explaining 29% of the positive effect on the primary composite end points of cardiovascular death, myocardial infarction, and stroke [31]. Inflammation and oxidative stress might be causes of higher mortality risks associated with hyperuricemia [20,32,33]. In healthy people, sodium-glucose-linked transporter 2 inhibitor with cardiovascular benefit was reported to increase urate excretion contrary to loop-diuretic [34], but current evidence could not tell us when and how starting ULT is helpful to CV risk in hyperuricemic adults. Although asymptomatic hyperuricemia was associated with increased risk of CKD [35,36], which has been found to increase all-cause and CVD mortality risks [37], ULT could not prevent kidneys from developing CKD with single-faceted treatment in people with normal renal function. In contrast, in patients with high low-density lipoprotein cholesterol, statin therapy can reduce mortality risk [38,39], no matter whether in diabetes or non-diabetes patients [40]. Management of risk factors of CKD progression and CVD in hyperuricemic adults should not only focus on reduction of serum UA level, but also on the potency of multifaceted risk control, similar to what clinicians have done for diabetes population.

Fang and Alderman have reported that ischemic heart disease mortality rate was 8.14/1000 person-years in males with a UA level of >7.0 mg/dL, who had no myocardial infarction, stroke, or gout at baseline [41]. In middle-aged men without history of CVD, followed up for 17 years, the CV mortality rate was 10.3/1000 person-years in participants with gout, implying an approximately 30% greater risk than those without gout (8.0/1000 person-years) [42]. In the trial comparing cardiovascular safety of febuxostat and allopurinol in patients with gout and a history of major cardiovascular disease, overall CV mortality rate of 14.2/1000 person-years was published [43]. In this study, the all-cause mortality and CV mortality in non-diabetes adults at a UA level of ≥9.0 mg/dL are 19.4/1000 person-years and 5.3/1000 person-years, respectively. The mortality risks are comparable to those with diabetes and a UA level of <7.0 mg/dL (20.4/1000 person-years and 5.1/1000 person-years). Exclusion of kidney disease and coronary history at baseline, as well as broader use of statins and angiotensin-converting-enzyme inhibitors/angiotensin II receptor blockers after the 2000s than in the 1990s, may be the causes of the lower proportion of CVD mortality in the present study. Although diabetes population had reduced CV mortality from >40% during 1999–2004 to approximately 20% during 2005–2010, the non-DM population had an increased proportion of cardiac death [12]. These results indicate that aggressive management of CV risk factors in hyperuricemic adults should be more important than only focusing on the effects of ULT. This study extends previous findings to further support that the mortality risk between diabetes and non-diabetes adults with high UA levels is similar, and so the same comprehensive treatment to manage other CV risk factors in both groups is reasonable.

Certain limitations deserve a mention. First, the time-varying changes in UA levels during the follow-up period of 6.6 years were not considered in this study. This was limited by the cross-sectional structure of NHANES data. In this kind of study, it is difficult to differentiate whether UA is just a marker or a leading cause of mortality. Second, the much smaller participant number in the diabetes group with a UA level of ≥9 mg/dL may produce biased change in mortality analyses across UA categories. Third, glycemic control may affect mortality risk, but glycated hemoglobin level was unable to be collected in all participants in NHANES data. Although indexes of glycemic control were not considered in this study, both CVD mortality and all-cause mortality were higher in participants with diabetes than in the non-diabetes group. One of the strengths of this study is due to it being the first study to the best of our knowledge, to compare the mortality risk across UA categories between diabetes and non-diabetes adults. Additionally, we completed the preliminary and multiethnic study to examine the mortality risk based on the nationally representative data in the United States. We utilized the large sample size to examine the relative HRs across subgroups where mortality risk is varying across UA categories. Furthermore, the participants without diabetes, CKD, and CHD in this study generally had the lowest mortality risk, and mortality risks in these adults are easily neglected in the setting of a clinical trial design. The epidemiological results in this study provided the material to examine the impact of hyperuricemia on these lowest-risk adults. 

In conclusion, we have shown that non-diabetes adults with a UA level of ≥7 mg/dL, who have not had CHD or CKD, have all-cause and CVD mortality risks similar to those among diabetes participants with a UA level of <7 mg/dL. This result may suggest that low-risk adults with hyperuricemia (UA level ≥ 7.0 mg/dL) could be managed as if they have DM. Adopting a more aggressive strategy to prevent mortality risk factors may be beneficial for adults with hyperuricemia. We propose that a stricter clinical trial to investigate the effects of comprehensive management of cardiovascular risk factors on hyperuricemic adults with lower risk should be conducted.

## 5. Conclusions

In summary, higher UA level of ≥7 mg/dL in the low-risk non-DM adults may indicate clinicians to address the multifaceted risk factors because their all-cause and CVD mortality risks would be similar to those with diabetes.

## Figures and Tables

**Table 1:** Baseline characteristics by diabetic status.

Variable	Overall	Non-DM	DM	*p*-Value ^a^
Number	29,226	26,720	2506	
Age, years	44.3 ± 0.2	43.6 ± 0.2	56.9 ± 0.4	<0.001
Male	13,736 (47.6)	12,560 (47.5)	1176 (46.7)	0.52
Non-Hispanic black	5876 (10.9)	5208 (10.4)	668 (16.8)	<0.001
Current smoker	6012 (23.0)	5565 (23.2)	447 (19.1)	<0.001
Cancer	2099 (7.4)	1805 (7.1)	294 (12.7)	<0.001
Hypertension	8075 (25.6)	6471 (23.2)	1604 (61.2)	<0.001
BMI, kg/m^2^	28.2 ± 0.1	27.9 ± 0.1	32.5 ± 0.2	<0.001
SBP	122 ± 0.2	121 ± 0.2	131 ± 0.7	<0.001
HDL-C, mg/dL	52.9 ± 0.2	53.2 ± 0.2	48.4 ± 0.4	<0.001
Fasting glucose, mg/dL	95.5 ± 0.2	91.6 ± 0.2	152.9 ± 1.9	<0.001
Total cholesterol, mg/dL	199.4 ± 0.4	199.7 ± 0.5	196.7 ± 1.4	<0.001
Triglycerides, mg/dL	147.4 ± 1.3	143.5 ± 1.2	205.2 ± 7.1	<0.001
Creatinine, mg/dL	0.84 ± 0.002	0.84 ± 0.002	0.85 ± 0.01	<0.001
DM duration, years	10.3 ± 0.3		10.3±0.3	

Abbreviations: BMI = body mass index; DM = diabetes mellitus; HDL-C = high-density lipoprotein cholesterol; SBP = systolic blood pressure. Continuous variables are expressed as mean ± standard error and categorical data are presented as numbers (percentage). ^a^ Comparison between non-DM and DM groups.

**Table 2:** Baseline characteristics by uric acid levels in non-DM and DM groups.

Variable	Non-DM				
	<5 (mg/dL)	5–7 (mg/dL)	7–9 (mg/dL)	≥9 (mg/dL)	*p*-Value ^a^
Number	11,545	11,895	3030	250	
Age, years	42.4 ± 0.2	44.3 ± 0.3	44.6 ± 0.4	45.3 ± 1.4	<0.001
Male	2306 (19.1)	7556 (63.8)	2498 (84.6)	200 (81.8)	
Non-Hispanic black	2106 (10.5)	2347 (10.2)	680 (10.4)	75 (17.5)	
Current smoker	2141 (22.1)	2746 (24.6)	630 (22.3)	48 (18.2)	
Cancer	700 (6.9)	869 (7.5)	220 (6.6)	16 (6.3)	
Hypertension	1961 (16.4)	3232 (26)	1140 (35.1)	138 (49.2)	<0.001
BMI, kg/m^2^	26.0 ± 0.1	28.8 ± 0.1	30.9 ± 0.2	31.6 ± 0.5	<0.001
SBP	118 ± 0.3	123 ± 0.2	126 ± 0.4	126 ± 1.4	<0.001
HDL-C, mg/dL	58.4 ± 0.3	50.4 ± 0.2	46.0 ± 0.3	45.9 ± 1.3	<0.001
Fasting Glucose, mg/dL	89.4 ± 0.2	92.6 ± 0.2	95.3 ± 0.4	96.0 ± 1.3	<0.001
Total cholesterol, mg/dL	195.9 ± 0.5	201.0 ± 0.7	206.6 ± 1.1	218.3 ± 3.5	<0.001
Triglycerides, mg/dL	118.5 ± 1.3	152 ± 1.6	193.2 ± 4.4	241.6 ± 22.4	<0.001
Creatinine, mg/dL	0.74 ± 0.002	0.89 ± 0.003	0.97 ± 0.005	1.01 ± 0.017	<0.001
	**DM**				
Number	973	1141	345	47	
Age, years	54.9 ± 0.6	57.9 ± 0.5	59.7 ± 0.9	54.5 ± 2.3	<0.001
Male	370 (37.5)	585 (50.5)	194 (57.5)	27 (63.1)	
Non-Hispanic black	204 (14.6)	299 (16.1)	142 (23.3)	23 (27.5)	
Current smoker	196 (21.4)	198 (19.8)	49 (12.4)	4 (7.5)	
Cancer	98 (12.4)	137 (12.6)	51 (14.2)	8 (12.0)	
Hypertension	510 (47.3)	781 (67.3)	268 (75.1)	45 (91.9)	<0.001
BMI, kg/m^2^	30.5 ± 0.3	33.3 ± 0.4	34.6 ± 0.5	36.3 ± 1.3	<0.001
SBP	130 ± 1.1	132 ± 0.9	132 ± 1.4	130 ± 3.9	<0.001
HDL-C, mg/dL	51.1 ± 0.7	47.7 ± 0.6	44.0 ± 0.7	40.7 ± 1.6	<0.001
Fasting Glucose, mg/dL	168.4 ± 3.8	144.6 ± 2.3	136.6 ± 3.8	158.0 ± 13.8	<0.001
Total cholesterol, mg/dL	197.6 ± 2.8	196.2 ± 1.9	196.3 ± 3.4	193.7 ± 9.6	<0.001
Triglycerides, mg/dL	190.5 ± 10.3	208.5 ± 12	226.8 ± 14.3	260 ± 47.2	<0.001
Creatinine, mg/dL	0.77 ± 0.006	0.88 ± 0.009	0.98 ± 0.017	1.05 ± 0.048	<0.001
DM duration, years	10.8 ± 0.5	9.9 ± 0.4	10.5±0.9	7.6±1.3	<0.001

Abbreviations: BMI = body mass index; DM = diabetes mellitus; HDL-C = high-density lipoprotein cholesterol; SBP = systolic blood pressure. Continuous variables are expressed as mean ± standard error and categorical data are presented as numbers (percentage). ^a^ *p* for trend across higher uric acid categories.

**Table 3:** Relative HRs (95% CI) compared to the group with UA level of 5–7 mg/dL for the association between mortality risks and uric acid levels among adults without diabetes and with diabetes.

Outcomes	Non-DM			
	<5 (mg/dL) *N* = 11,545	5–7 (mg/dL) *N* = 11,895	7–9 (mg/dL) *N* = 3030	≥9 (mg/dL) *N* = 250
All-cause mortality (*N* = 1664)				
un-weighted	1.02 (0.8–1.18)	1.0 (reference)	1.26 (1.06–1.49) *	2.44 (1.63–3.66) *
weighted ^a^	1.03 (0.87–1.22)	1.0 (reference)	1.43 (1.15–1.79) *	2.41 (1.42–4.08) *
CVD mortality (*N* = 378)				
un-weighted	1.06 (0.78–1.44)	1.0 (reference)	1.37 (0.96–1.95)	2.97 (1.37–6.45) *
weighted ^a^	1.22 (0.82-1.82)	1.0 (reference)	1.76 (1.22–2.56) *	5.06 (1.69–15.15) *
Cancer death (*N* = 436)				
un-weighted	1.06 (0.82-1.36)	1.0 (reference)	1.09 (0.79–1.49)	2.39 (1.17–4.90) *
weighted ^a^	1.04 (0.75-1.45)	1.0 (reference)	1.25 (0.90–1.73)	1.43 (0.64–3.21)
CVD or Cancer death (*N* = 814)				
un-weighted	1.06 (0.87-1.29)	1.0 (reference)	1.19 (0.94–1.51)	2.63 (1.56–4.45) *
weighted ^a^	1.10 (0.84-1.43)	1.0 (reference)	1.42 (1.08–1.87) *	2.60 (1.21–5.58) *
	**DM**			
	***N* = 973**	***N* = 1141**	***N* = 345**	***N* = 47**
All-cause mortality (*N* = 405)				
un-weighted	1.33 (1.01–1.75) *	1.0 (reference)	1.69 (1.22–2.34) *	1.41 (0.65–3.08)
weighted ^a^	1.66 (1.14–2.41) *	1.0 (reference)	2.17 (1.49–3.17) *	2.10 (0.87–5.06)
CVD mortality (*N* = 117)				
un-weighted	0.71 (0.41–1.24)	1.0 (reference)	1.75 (0.95–3.23)	1.91 (0.45–8.11)
weighted ^a^	0.91 (0.38–2.18)	1.0 (reference)	2.53 (1.18–5.41) *	0.89 (0.18–4.50)
Cancer death (*N* = 84)				
un-weighted	1.82 (1.01–3.26) *	1.0 (reference)	1.81 (0.85–3.85)	2.60 (0.59–11.40)
weighted ^a^	2.13 (0.84–5.42)	1.0 (reference)	2.13 (0.88–5.12)	4.51 (0.89-22.78)
CVD or Cancer death (*N* = 201)				
un-weighted	1.10 (0.74–1.63)	1.0 (reference)	1.75 (1.09–2.81)	2.15 (0.77–6.04)
weighted ^a^	1.36 (0.77–2.43)	1.0 (reference)	2.30 (1.34–3.96) *	2.60 (0.69–9.80)

^a^ Data are weighted estimates. Adjusted for BMI, sex, age, race, HDL-Cholesterol, current smoking status, SBP and creatinine. * *p* < 0.05.

**Table 4:** Relative HRs (95% CI) of mortality risks compared with non-diabetes participants with UA 5–7 mg/dL among participants without diabetes and with diabetes.

**Risks**	**Non-DM**				**DM**			
	**<5 (mg/dL)** ***N* = 11,545**	**5–7 (mg/dL)** ***N* = 11,895**	**7–9 (mg/dL)** ***N* = 3030**	**≥9 (mg/dL)** ***N* = 250**	**<5 (mg/dL)** ***N* = 973**	**5–7 (mg/dL)** ***N* = 1141**	**7–9 (mg/dL)** ***N* = 345**	**≥9 (mg/dL)** ***N* = 47**
**All-cause mortality (*N* = 2069)**								
un-weighted	1.04 (0.9–1.19)	1.0 (reference)	1.24 (1.05–1.47) *	2.37 (1.58–3.55) *	1.77 (1.44–2.18) *	1.43 (1.17–1.76) *	2.55 (1.94–3.34) *	2.25 (1.06–4.77) *
weighted ^a^	1.06 (0.9–1.26)	1.0 (reference)	1.40 (1.12–1.75) *	2.35 (1.39–3.96) *	2.01 (1.55–2.62) *	1.30 (0.95–1.76)	2.87 (2.14–3.85) *	2.79 (1.28–6.08) *
**CVD mortality (*N* = 495)**								
un-weighted	1.03 (0.77–1.39)	1.0 (reference)	1.39 (0.98–1.98)	3.06 (1.42–6.62) *	1.71 (1.08–2.69) *	2.12 (1.46–3.09) *	3.25 (1.89-5.61) *	3.38 (0.82–13.87)
weighted ^a^	1.23 (0.86–1.76)	1.0 (reference)	1.74 (1.18–2.57) *	5.07 (1.71–5.04) *	2.21 (1.14–4.28) *	2.25 (1.25–4.06) *	4.99 (2.48–10.03) *	1.66 (0.42–6.60)
**Cancer death (*N* = 520)**								
un-weighted	1.04 (0.81–1.34)	1.0 (reference)	1.10 (0.80–1.50)	2.42 (1.18–4.94) *	1.58 (1.07–2.33) *	0.82 (0.52–1.30)	1.28 (0.69–2.39)	1.84 (0.45–7.47)
weighted ^a^	1.07 (0.77–1.49)	1.0 (reference)	1.23 (0.88-1.71)	1.43 (0.64–3.19)	1.45 (0.77–2.72)	0.78 (0.45–1.35)	1.54 (0.75–3.17)	3.30 (0.76–14.37)
**CVD or Cancer death (*N* = 1015)**								
un-weighted	1.04 (0.86–1.26)	1.0 (reference)	1.22 (0.96–1.54)	2.67 (1.58–4.51) *	1.62 (1.21–2.18) *	1.35 (1.02–1.80) *	2.01 (1.34–3.02) *	2.40 (0.89–6.50)
weighted ^a^	1.13 (0.86–1.47)	1.0 (reference)	1.40 (1.06–1.84) *	2.58 (1.21–5.54) *	1.69 (1.14–2.51) *	1.28 (0.89–1.84)	2.67 (1.68–4.26) *	2.81 (0.84–9.39)

^a^ Data are weighted estimates. Adjusted for BMI, sex, age, race, HDL-Cholesterol, current smoking status, SBP and creatinine. * *p* < 0.05.

## Supplementary Materials

The following are available online at https://www.mdpi.com/2077-0383/8/12/2127/s1, Figure S1: Flowchart of enrollment of study population, Table S1: Stepwise analysis of relative HRs (95%CI) for the association between mortality risks and uric acid levels among adults without diabetes and with diabetes., Table S2: Stepwise analysis of relative HRs (95%CI) of mortality risks compared with non-diabetes participants with UA 5–7 mg/dL among participants without diabetes and with diabetes. Table S3: Racial differences in relative HRs (95%CI) of mortality risks compared with non-diabetes participants with UA 5–7 mg/dL.
