# Supplementary material for: Approximate Mortality Risks between Hyperuricemia and Diabetes in the United States
Source: J Clin Med. 2019 Dec 3;8(12):2127. doi: 10.3390/jcm8122127 (PMC6947281; doi:10.3390/jcm8122127)
Supplement: Supplementary file 1 [file jcm-08-02127-s001.pdf]

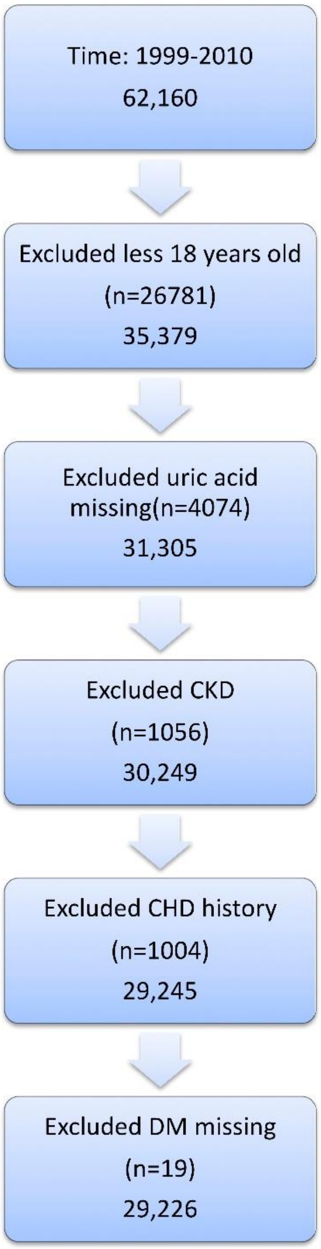

Figure S1. Flow chart of enrollment.

Supplementary table 1. Stepwise analysis of relative HRs (95%CI) for the association between mortality risks and uric acid levels among adults without diabetes and with diabetes.

	Non-DM			
	<5 (mg/dL)<br>N =11545	5-7<br>N =11895	7-9<br>N =3030	≥9<br>N =250
All-cause mortality (N =1664)				
univariate	0.79(0.68-0.91)	1.0(reference)	1.33(1.10-1.62)*	2.67(1.64-4.36)*
Model1	1.01(0.87-1.18)	1.0(reference)	1.37(1.10-1.71)*	2.79(1.75-4.45)*
Model2	1.03(0.87-1.22)	1.0(reference)	1.43(1.15-1.79)*	2.41(1.42-4.08)*
CVD mortality (N =378)				
univariate	0.91(0.70-1.17)	1.0(reference)	1.70(1.25-2.32)*	4.22(1.67-10.65)*
Model1	1.14(0.78-1.67)	1.0(reference)	1.73(1.18-2.52)*	4.69(1.76-12.46)*
Model2	1.22(0.82-1.82)	1.0(reference)	1.76(1.22-2.56)*	5.06(1.69-15.15)*
Cancer death(N =436)				
univariate	0.78(0.59-1.05)	1.0(reference)	1.29(0.97-1.71)	1.81(0.92-3.53)
Model1	1.09(0.79-1.51)	1.0(reference)	1.18(0.86-1.63)	1.65(0.86-3.19)
Model2	1.04(0.75-1.45)	1.0(reference)	1.25(0.9-1.73)	1.43(0.64-3.21)
CVD or Cancer death (N =814)				
univariate	0.83(0.68-1.02)	1.0(reference)	1.45(1.15-1.82)*	2.73(1.40-5.32)*
Model1	1.11(0.86-1.43)	1.0(reference)	1.37(1.04-1.8)*	2.62(1.36-5.05)*
Model2	1.10(0.84-1.43)	1.0(reference)	1.42(1.08-1.87)*	2.60(1.21-5.58)*
	DM			
	N =973	N =1141	N =345	N =47
All-cause mortality (N =405)				
univariate	1.21(0.87-1.69)	1.0(reference)	2.33(1.66-3.27)*	2.04(0.92-4.53)
Model1	1.58(1.12-2.24)*	1.0(reference)	2.20(1.51-3.20)*	1.96(0.84-4.55)
Model2	1.66(1.14-2.41)*	1.0(reference)	2.17(1.49-3.17)*	2.10(0.87-5.06)
CVD mortality (N =117)				
univariate	0.81(0.44-1.48)	1.0(reference)	2.48(1.37-4.47)*	0.49(0.10-2.45)
Model1	0.87(0.36-2.12)	1.0(reference)	2.43(1.25-4.76)*	0.66(0.14-3.18)
Model2	0.91(0.38-2.18)	1.0(reference)	2.53(1.18-5.41)*	0.89(0.18-4.50)
Cancer death (N =84)				
univariate	1.28(0.58-2.82)	1.0(reference)	1.88(0.94-3.78)	4.80(1.43-16.05)*
Model1	2.22(0.86-5.76)	1.0(reference)	2.10(0.94-4.66)	4.53(0.98-20.96)
Model2	2.13(0.84-5.42)	1.0(reference)	2.13(0.88-5.12)	4.51(0.89-22.78)
CVD or Cancer death (N =201)				
univariate	1.00(0.62-1.62)	1.0(reference)	2.24(1.41-3.56)*	2.32(0.81-6.65)

Model1	1.36(0.78-2.38)	1.0(reference)	2.31(1.40-3.81)*	2.15(0.63-7.36)
Model2	1.36(0.77-2.43)	1.0(reference)	2.30(1.34-3.96)*	2.60(0.69-9.8)

[a]Data are weighted estimates.

Model1. Adjusted for BMI, sex, age, race, current smoking status. *$P < 0.05$.

Model2. Adjusted for BMI, sex, age, race, HDL-Cholesterol, current smoking status, SBP and Creatinine . *$P < 0.05$.

Supplementary table 2. Stepwise analysis of relative HRs (95%CI) of mortality risks compared with non-diabetes participants with UA 5-7mg/dL among participants without diabetes and with diabetes.

	**Non-DM**				**DM**			
	**<5 (mg/dL) N =11545**	**5-7 N =11895**	**7-9 N =3030**	**≥9 N =250**	**<5 (mg/dL) N =973**	**5-7 N =1141**	**7-9 N =354**	**≥9 N =47**
All-cause mortality (N =2069)								
univariate	0.79(0.68-0.91)	1.0(reference)	1.33(1.1-1.62)*	2.67(1.64-4.36)*	3.18(2.45-4.13)*	2.63(1.98-3.49)*	6.12(4.55-8.25)*	5.37(2.53-11.39)*
Model1	1.04(0.89-1.21)	1.0(reference)	1.35(1.08-1.69)*	2.75(1.73-4.37)*	1.98(1.53-2.57)*	1.31(0.97-1.78)	2.95(2.22-3.92)*	2.57(1.18-5.6)*
Model2	1.06(0.9-1.26)	1.0(reference)	1.4(1.12-1.75)*	2.35(1.39-3.96)*	2.01(1.55-2.62)*	1.3(0.95-1.76)	2.87(2.14-3.85)*	2.79(1.28-6.08)*
CVD mortality (N =495)								
univariate	0.9(0.7-1.17)	1.0(reference)	1.7(1.25-2.33)*	4.21(1.66-10.64)*	3.71(2.04-6.76)*	4.56(2.92-7.11)*	11.29(6.66-19.14)*	2.13(0.46-10.01)
Model1	1.18(0.83-1.67)	1.0(reference)	1.7(1.16-2.51)*	4.58(1.71-12.24)*	1.98(1.01-3.88)*	2.24(1.27-3.96)*	5.28(2.87-9.73)*	1.41(0.33-5.98)
Model2	1.23(0.86-1.76)	1.0(reference)	1.74(1.18-2.57)*	5.07(1.71-15.04)*	2.21(1.14-4.28)*	2.25(1.25-4.06)*	4.99(2.48-10.03)*	1.66(0.42-6.6)
Cancer death (N =520)								
univariate	0.78(0.59-1.05)	1.0(reference)	1.29(0.97-1.72)	1.81(0.93-3.54)	2.38(1.36-4.19)*	1.9(1.11-3.23)*	3.54(1.9-6.59)*	9.31(3.03-28.58)*
Model1	1.13(0.82-1.55)	1.0(reference)	1.17(0.85-1.61)	1.66(0.86-3.2)	1.56(0.86-2.84)	0.79(0.46-1.34)	1.55(0.76-3.16)	3.29(0.78-13.92)
Model2	1.07(0.77-1.49)	1.0(reference)	1.23(0.88-1.71)	1.43(0.64-3.19)	1.45(0.77-2.72)	0.78(0.45-1.35)	1.54(0.75-3.17)	3.3(0.76-14.37)
CVD or Cancer death (N =1015)								
univariate	0.83(0.68-1.02)	1.0(reference)	1.45(1.15-1.82)*	2.73(1.4-5.32)*	2.89(1.95-4.27)*	2.9(2.06-4.09)*	6.49(4.33-9.71)*	6.62(2.45-17.89)*
Model1	1.14(0.88-1.47)	1.0(reference)	1.35(1.03-1.77)*	2.61(1.35-5.03)*	1.69(1.16-2.47)*	1.3(0.91-1.85)	2.89(1.87-4.46)*	2.64(0.81-8.61)
Model2	1.13(0.86-1.47)	1.0(reference)	1.4(1.06-1.84)*	2.58(1.21-5.54)*	1.69(1.14-2.51)*	1.28(0.89-1.84)	2.67(1.68-4.26)*	2.81(0.84-9.39)

[a]Data are weighted estimates.

Model1. Adjusted for BMI, gender, age, race, current smoking status. *$P < 0.05$.

Model2. Adjusted for BMI, gender, age, race, HDL-Cholesterol, current smoking status, SBP and Creatinine . *$P < 0.05$.

Supplementary Table 3-1. Relative HRs (95%CI) of mortality risks compared with non-diabetes participants with UA 5-7mg/dL among non-Hispanic white participants without diabetes and with diabetes.

Non-Hispanic White	Non-DM				DM			
	**<5 (mg/dL) N =5312**	**5-7 N =5795**	**7-9 N =1522**	**≥9 N =98**	**<5 (mg/dL) N =312**	**5-7 N =424**	**7-9 N =135**	**≥9 N =15**
All-cause mortality (N=1185)	1.11(0.90-1.37)	1.0(reference)	1.49(1.15-1.94)*	2.32(1.07-5.01)*	1.99(1.46-2.72)*	1.19(0.83-1.71)	3.37(2.35-4.84)*	4.32(1.81-10.34)*
CVD mortality (N=276)	1.43(0.92-2.22)	1.0(reference)	1.68(1.04-2.70)*	7.05(1.9-26.09)*	2.49(1.15-5.36)*	1.34(0.60-3.01)	6.63(3.08-14.26)*	No event
Cancer death (N=289)	1.13(0.77-1.66)	1.0(reference)	1.47(0.99-2.19)	0.44(0.06-3.00)	1.46(0.59-3.60)	0.91(0.46-1.82)	2.11(0.88-5.09)	5.82(1.27-26.68)*
CVD or Cancer death (N=565)	1.23(0.90-1.69)	1.0(reference)	1.54(1.09-2.19)*	2.48(0.81-7.57)	1.80(1.09-2.97)*	1.06(0.64-1.74)	3.73(2.15-6.46)*	4.02(0.92-17.53)

[a]Data are weighted estimates. Adjusted for BMI, gender, age, HDL-Cholesterol, current smoking status, SBP and creatinine. *$P < 0.05$.

Supplementary Table 3-2. Relative HRs (95%CI) of mortality risks compared with non-diabetes participants with UA 5-7mg/dL among non-Hispanic black participants without diabetes and with diabetes.

Non-Hispanic Black	Non-DM				DM			
	**<5 (mg/dL) N =2106**	**5-7 N =2347**	**7-9 N =680**	**≥9 N =75**	**<5 (mg/dL) N =204**	**5-7 N =299**	**7-9 N =142**	**≥9 N =23**
All-cause mortality (N=412)	0.68(0.45-1.04)	1.0(reference)	1.31(0.91-1.9)	3.21(1.47-7.04)*	1.84(1.12-3.03)*	1.37(0.91-2.04)	2.62(1.67-4.09)*	0.96(0.29-3.22)
CVD mortality (N=92)	0.52(0.22-1.21)	1.0(reference)	1.32(0.61-2.86)	1.68(0.28-10.03)	1.70(0.55-5.24)	2.50(1.07-5.87)*	1.84(0.42-8.10)	2.31(0.59-9.00)
Cancer death (N=117)	0.69(0.31-1.53)	1.0(reference)	0.76(0.45-1.27)	3.26(1.25-8.48)*	1.49(0.60-3.7)	0.27(0.08-0.88)	0.84(0.44-1.62)	No event
CVD or Cancer death (N=209)	0.64(0.34-1.20)	1.0(reference)	0.91(0.58-1.44)	2.68(0.89-8.09)	1.53(0.77-3.04)	0.99(0.52-1.89)	1.12(0.53-2.37)	0.83(0.15-4.53)

[a]Data are weighted estimates. Adjusted for BMI, gender, age, HDL-Cholesterol, current smoking status, SBP and creatinine. *$P < 0.05$.

Supplementary Table 3-3. Relative HRs (95%CI) of mortality risks compared with non-diabetes participants with UA 5-7mg/dL among participants of other races without diabetes and with diabetes.

Other Races	**Non-DM**				**DM**			
	**<5 (mg/dL) N =4127**	**5-7 N =3753**	**7-9 N =828**	**≥9 N =77**	**<5 (mg/dL) N =457**	**5-7 N =418**	**7-9 N =68**	**≥9 N =9**
All-cause mortality (N=472)	1.26(0.87-1.84)	1.0(reference)	0.99(0.53-1.84)	0.58(0.16-2.13)	2.12(1.17-3.86)*	1.55(0.81-2.95)	1.70(0.59-4.91)	1.48(0.16-13.98)
CVD mortality (N=127)	1.49(0.56-3.96)	1.0(reference)	3.20(1.21-8.47)*	0.79(0.09-6.93)	1.64(0.51-5.26)	8.19(2.66-25.22)*	0.54(0.06-4.64)	32.52(2.41-438.30)*
Cancer death (N=114)	1.18(0.41-3.43)	1.0(reference)	0.20(0.05-0.74)	1.40(0.26-7.47)	1.16(0.32-4.23)	0.77(0.24-2.47)	0.42(0.05-3.47)	No event
CVD or Cancer death (N=241)	1.26(0.6-2.63)	1.0(reference)	1.05(0.42-2.58)	1.27(0.3-5.35)	1.31(0.50-3.40)	2.76(1.19-6.40)*	0.46(0.09-2.31)	7.14(0.78-65.34)

[a]Data are weighted estimates. Adjusted for BMI, gender, age, HDL-Cholesterol, current smoking status, SBP and creatinine. *P < 0.05.
